# The *Retinoblastoma-related gene RBL901* can trigger drought response actions in potato

**DOI:** 10.1007/s00299-023-03055-0

**Published:** 2023-08-09

**Authors:** Dorota Sołtys-Kalina, Alicja Macko-Podgórni, Jarosław Plich, Dominika Boguszewska-Mańkowska, Katarzyna Szajko, Paulina Smyda-Dajmund, Dariusz Grzebelus, Waldemar Marczewski

**Affiliations:** 1 Plant Breeding and Acclimatization Institute–National Research Institute in Radzików, Młochów Division, Platanowa Str. 19, 05-831 Młochów, Poland; 2 Department of Plant Biology and Biotechnology, Faculty of Biotechnology and Horticulture, University of Agriculture in Krakow, 31-120 Krakow, Poland; 3 Plant Breeding and Acclimatization Institute–National Research Institute in Radzików, Jadwisin Division, Szaniawskiego Str. 15, 05-140 Jadwisin, Poland

**Keywords:** Cell cycle progression, Drought, Gene expression, Potato, Yield

## Abstract

This study provided new insights into response of potato to drought stress.

**Supplementary Information:**

The online version contains supplementary material available at 10.1007/s00299-023-03055-0.

The cultivated potato (*Solanum tuberosum* L.) is one of the most important crops in the world. It is estimated that, globally, over the next few decades, drought will decrease potential potato yields and quality, which are the main determinants of potato tolerance to drought stress. Potato plants are sensitive to drought stress, although cultivar-dependent variability in the response to water deficiency has been observed (Nasir and Toth 2022). In our previous studies, a 3-week drought treatment decreased tuber yield in a half-sib family of Katahdin-derived potato cultivars (Sołtys-Kalina et al. 2016; Plich et al. 2020). In the present experiments, a 2-week period of drought stress was probably too short to cause statistically significant differences in the mean tuber yield per plant in the cultivars Cayuga, Dalila, Katahdin, Pontiac, Sebago, and Seneca (Table S1). However, this stress significantly reduced average tuber weight (ATW) in the cultivars Katahdin and Pontiac. ATW was significantly lower (p value ≤ 0,001) in the drought-stressed plants (R, recovered plants) of the cultivars Katahdin (26% decrease) and Pontiac (31% decrease) in relation to the control plants (C) (Fig. 1a). This may imply a decrease in tuber marketability for the cultivars Katahdin and Pontiac. Tubers harvested from C and R plants of the cultivar Katahdin are shown in Fig. 1b.

**Fig. 1:**
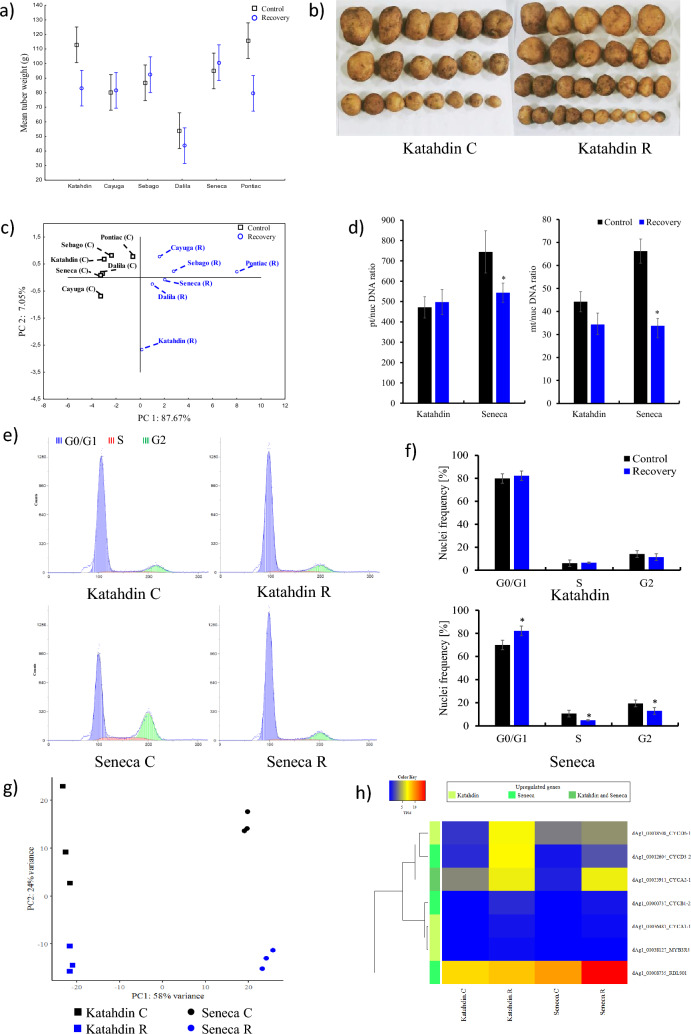
Characterization of the potato response to drought in control plants (C) and plants recovered from drought stress (R). **a** Average tuber weight. **b** Tubers of control (C) and recovered (R) plants of the cultivar Katahdin. **c** Biplot of principal component analysis (PCA) scores of PC1 vs. PC2 of six potato cultivars based on selected JIP parameters. **d** Left panel –plastid/nuclear DNA ratios (pt/nucDNA), right panel–mitochondrial/nuclear DNA (mt/nucDNA) ratios of the cultivars Katahdin and Seneca (means, ± SD). **e** Representative flow cytometry histograms derived from the nucleus distribution within the cell cycle of Katahdin and Seneca. **f** Graphs of nucleus frequency based on independent cell cycle experiments (means, ± SD). **g** PCA plot based on the normalized expression of differentially expressed genes (DEGs) in the cultivars Katahdin and Seneca. **h** Heatmap of normalized expression values (transcripts per million, TPM) of cell cycle-related DEGs in the cultivars Katahdin and Seneca. *- Student’s t-test

Potato tubers are strong sink organs. Photosynthesis in leaves provides the supplies needed for biomass accumulation. Understanding the biological basis of decreases in potato tuber yields and quality in response to water shortage, considering the responses of leaves, is a serious agronomic challenge (Boguszewska-Mańkowska et al. 2020). The canopy response to drought was analysed based on the JIP test, measuring fluorescence parameters that indicate the efficiencies and fluxes of electrons and energy around PSI and PSII in plants subject to stress events (Boguszewska-Mańkowska et al. 2020). Twelve JIP parameters showed statistically significant differences between C and R plants (Table S2). In Katahdin, the value of the JIP parameter PI_total_ reached 122% of that in the control, whereas a decrease in that parameter was observed in other cultivars. PI_total_ is used as an indicator of drought (Viljevac Vuletić et al. 2022, and references therein). Principal component analysis (PCA) performed based on the main components, PC1 and PC2, separated R plants of the cultivars Pontiac and Katahdin as showing the most distinct responses to drought (Fig. 1c). Based on ATW and PCA, Katahdin and Seneca (both late-maturing cultivars) were selected as the models for the organelle/nuclear DNA ratio, cell cycle progression and gene expression comparisons.

In plants, the degradation and recycling of unnecessary cytosolic structures (e.g. plastids and mitochondria) maintain cellular homeostasis under normal conditions and are upregulated during a wide range of abiotic/biotic stresses (Su et al. 2020). In our study, R plants of the cultivar Seneca showed a plastid/nuclear DNA ratio (pt/nucDNA) that was 1.96-fold (*P* < 0.001) lower than that of the C plants. For the mitochondrial/nuclear DNA (mt/nucDNA) ratio, the corresponding value for Seneca was 1.37-fold (*P* < 0.001) lower. No significant differences were observed in Katahdin (Fig. 1d). We postulate that the drought-induced autophagic machinery was expressed more efficiently in the cultivar Seneca.

There is a close association between cell cycle progression and plant adaptation to drought stress (Qi and Zhang 2020). The primary response of plants to stress events is the inhibition of growth. The balance between cell cycle progression and adaptation (e.g., growth inhibition) to dynamic environmental conditions is critical for adaptive success. In the leaves of Katahdin and Seneca that had recovered from drought, cell cycle progression was evaluated in the middle part of the lamina and compared to the reaction in control plants. In the cultivar Seneca, cell cycle progression was modulated in response to drought stress, whereas no significant changes in the cell cycle were observed in Katahdin (Fig. 1e). These modifications were related to a decreasing frequency of nuclei in S phase (54.3%) and an increased frequency of nuclei in G0/G1 phase (17.5%). A decrease in the frequency of nuclei in G2 phase (33.3%) was also noted (Fig. 1f). The cell cycle arrest in G0/G1 phase observed in the cultivar Seneca can be interpreted as a component of plant adaptation to drought. This delay in cell cycle progression allows the repair of DNA damage and the initiation of programmed cell death or senescence to avoid unfavourable changes in progeny cells in response to stress treatment.

Differentially expressed genes (DEGs) were identified in the cultivars Katahdin and Seneca. DEGs (*P* value < 0.05) were selected by comparing the corresponding C and R samples (Tables S3-S6). We identified higher numbers of both up- and downregulated genes in the leaves of Seneca (1665 and 1106, respectively) than in the leaves of Katahdin (416 and 155, respectively) (Figure S1). PCA showed that the transcriptional profile was more dependent on the plant cultivar than on the treatment. PC1 clearly separated the cultivars and explained 58% of the variance, while PC2 explained 24% of the variance and separated the control and drought-stressed plants. PCA indicated a closer relationship of Katahdin plants grown under control and drought conditions compared to Seneca plants, for which the grouping of plants representing different experimental conditions was more prominent (Fig. 1g). Subsequently, we focused on cell cycle-related genes (Figure S2). Among these genes, three were significantly upregulated only in Katahdin, three only in Seneca, and one in both cultivars (Fig. 1h), while no downregulated genes were revealed. Two of the seven DEGs were classified as D-type cyclins (*CYCD6-1*, *CYCD3-2*), two were classified as A-type cyclins (*CYCA1-1*, *CYCA2-1*) and the remaining gene *CYCB1-2* belonged to the B-type cyclins. These genes are downstream targets of cyclin-dependent kinases (CDKs) that coordinate cell cycle progression (Carneiro et al. 2021; Qi and Zhang 2020; Saidi and Hajibarat 2021). In plants, MYB3R4 transcription factors regulate the expression of G2/M-specific genes (Carneiro et al. 2021). In our study, the transcript level of one *MYB3R4* gene was upregulated in R plants of the cultivar Katahdin (Fig. 1h). Retinoblastoma-related protein plays a multifunctional role in cell cycle regulation. The proteins encoded by *Retinoblastoma-Related* (*RBR*) genes are negative regulators of the G1/S transition (Banerjee et al. 2020). Here, the *RBR* homologue *RBL901* was one of four genes related to the regulation of the cell cycle that showed significantly higher expression in R plants of Seneca than in the corresponding C plants, while in Katahdin, its expression was not significantly changed by drought stress (Fig. 1h). It should be emphasized that this gene presented the highest expression among all DEGs encoding proteins controlling cell cycle progression, a 1.7-fold increase in expression in Seneca R plants (Fig. 1h). In summary, we report for the first time that cell cycle progression in the leaves of drought-stressed potato plants likely plays a significant role in the final tuber yield distribution. We found that the level of the *RBL901* transcript was related to the cell cycle progression data, where the increase in the frequency of nuclei in G0/G1 phase was accompanied by a decreased frequency of nuclei in S phase. We postulate that *RBL901* might be a novel candidate factor involved in the regulation of the potato response to drought stress.

## Supplementary Information

Below is the link to the electronic supplementary material.

Supplementary file1 (DOCX 18 KB)

Supplementary file2 (DOCX 1951 KB)

Supplementary file3 (DOCX 26 KB)

Supplementary file4 (XLSX 214 KB)

## Data Availability

All raw and processed data have been deposited in the SRA database (Sequence Read Archive) under the link: https://www.ncbi.nlm.nih.gov/bioproject/956351.
