# Supplementary material for: The Retinoblastoma-related gene RBL901 can trigger drought response actions in potato
Source: Plant Cell Rep. 2023 Aug 9;42(10):1701–4. doi: 10.1007/s00299-023-03055-0 (PMC10505103; doi:10.1007/s00299-023-03055-0)
Supplement: Supplementary file 1 — Supplementary file1 (DOCX 18 KB) [file 299_2023_3055_MOESM1_ESM.docx]

Plant material

The cultivar Katahdin and 5 Katahdin-derived potato cultivars were obtained from the potato collection of the Plant Breeding and Acclimatization Institute-National Research Institute,

Bonin, Poland. The cultivars were released by potato breeding companies in the USA (cvs. Cayuga, Katahdin, Pontiac, Sebago and Seneca) and Poland (cv. Dalila).

Yield analysis

The experiments were carried out in 2021. Plants were first sprouted and grown in the greenhouse and then planted in a tent as described in Sołtys-Kalina *et al*. (2016). Two treatments were performed. In the irrigation treatment, water was applied to plants that were still irrigated optimally (C, control plants). In the drought treatment, irrigation was stopped 30 days after planting for 2 weeks (R, recovery plants). The experiment was run in a randomized complete block design with three blocks (replications) and three plants per block. In total, 18 plants per cultivar (2 treatments, 3 blocks per treatment, and 3 plants per block) were tested. Tubers were harvested 135 days after planting. The mean values of the total tuber yield per plant and average tuber weight per plant were calculated for each replicate. Statistical analysis was performed as described in Plich *et al*. 2020.

Analysis of fluorescence parameters

Measurements were performed for C and R plants (3 days after drought release) as described in Plich *et al*. 2020. For each sample, 6 biological replications were scored.

DNA extraction and qPCR for the determination of organelle DNA content

DNA quality and quantity were assessed as described in Niu et al. (2019). Organelle DNA content was evaluated in leaf samples from C and R plants. For each sample, 6 biological replications and 3 technical repetitions were scored. The protocol details are described in Szajko *et al*. 2022. For each sample, 6 biological replications were scored.

Cell cycle analysis

The cell cycle was analysed using a CyFlow flow cytometer (Sysmex, Polska) equipped with a UV diode. Cell nuclei were isolated from the middle part of the leaves of control (C) and recovery (R) plants using CyStain UV OxProtect Sysmex, Polska) isolation buffer with DAPI stain. For each sample, the fluorescence of 30 000 nuclei was analysed. Histograms were evaluated using FlowMax 3.1x software (Sysmex, Polska) using the Cell Cycle Analysis panel. The results are expressed as the percentage of nuclei in G0/G1, S and G2 phases. For each sample, 6 biological replications were scored.

Gene expression studies

RNA was isolated from leaves of control (C) and recovery (R) plants in three biological replications using a Sigma RNA isolation kit according to the manufacturer’s protocol. RNA quality (RIN>8) was measured on a Bioanalyzer 2100 Agilent. RNAseq experiments were performed by Genomed S.A. (Poland). RNAseq was sequenced using the Illumina NovaSeq 6000 sequencing platform. Transcriptomic data were analysed according to Machaj *et al*. (2018). For each sample, 3 biological replications were scored. All cleaned reads have been deposited in the Sequence Read Archive (SRA) database under BioProject PRJNA956351.

References

Machaj, G., Bostan, H., Macko-Podgórni, A., Iorizzo, M., & Grzebelus, D. (2018). Comparative transcriptomics of root development in wild and cultivated carrots. Genes, 9(9), 431.  https://doi.org/[10.3390/genes9090431](https://doi.org/10.3390%2Fgenes9090431)

Niu, S; Zhang, G; Li, X; Haroon, M; Si, H; Fan, G; Li, X-Q. (2019) T Organelle DNA contents and starch accumulation in potato tubers *Theor Appl Genet* 132:205-216.

Plich, J.; Boguszewska-Mańkowska, D. and Marczewski, W. (2020) Relations Between Photosynthetic Parameters and Drought-Induced Tuber Yield Decrease in Katahdin-Derived Potato Cultivars. *Potato Res*. **63**, 463–477.

Sołtys-Kalina, D; Plich, J; Strzelczyk-Żyta, D; Śliwka, J and Marczewski, W. (2016) The effect of drought stress on the leaf relative water content and tuber yield of a half-sib family of ‘Katahdin’-derived potato cultivars. *Breed Sci* 66:328–331.

Szajko, K; Sołtys-Kalina, D; Heidorn-Czarna, M; Smyda-Dajmund, P; Wasilewicz-Flis, I; Jańska, H. and Marczewski, W. (2022) Transcriptomic and proteomic data provide new insights into cold-treated potato tubers with T- and D- type cytoplasm. *Planta* 255:97.
