# Supplementary material for: The Retinoblastoma-related gene RBL901 can trigger drought response actions in potato
Source: Plant Cell Rep. 2023 Aug 9;42(10):1701–4. doi: 10.1007/s00299-023-03055-0 (PMC10505103; doi:10.1007/s00299-023-03055-0)
Supplement: Supplementary file 2 — Supplementary file2 (DOCX 1951 KB) [file 299_2023_3055_MOESM2_ESM.docx]

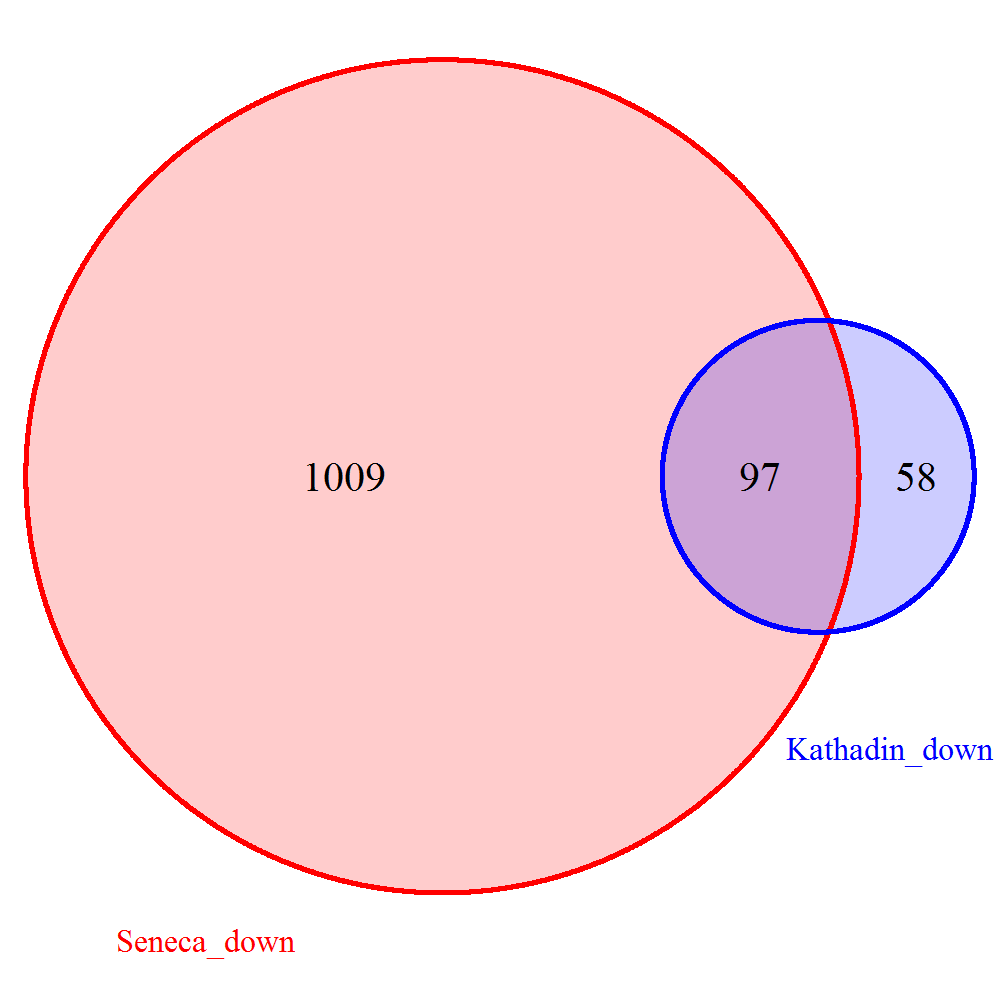

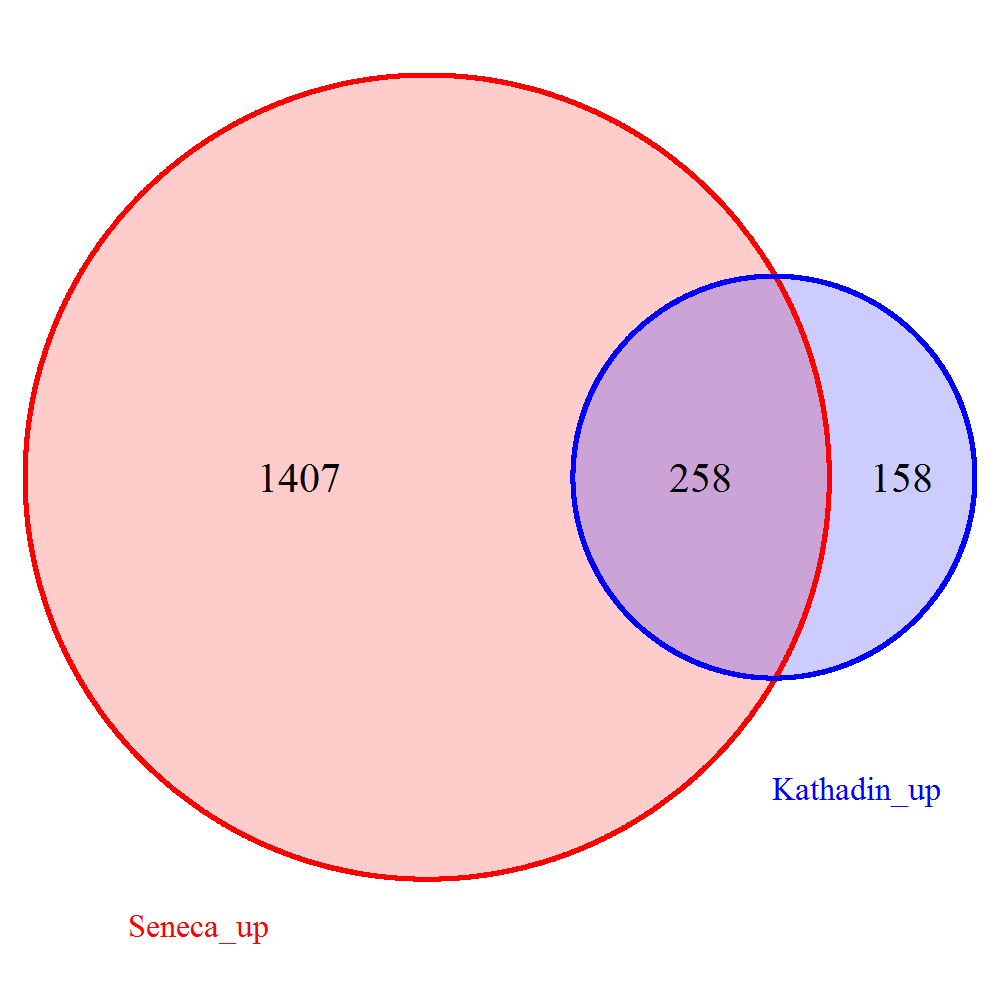


Figure S1. Venn diagrams representing the number of differentially expressed genes (DEGs) identified in cultivars Katahdin and Seneca.


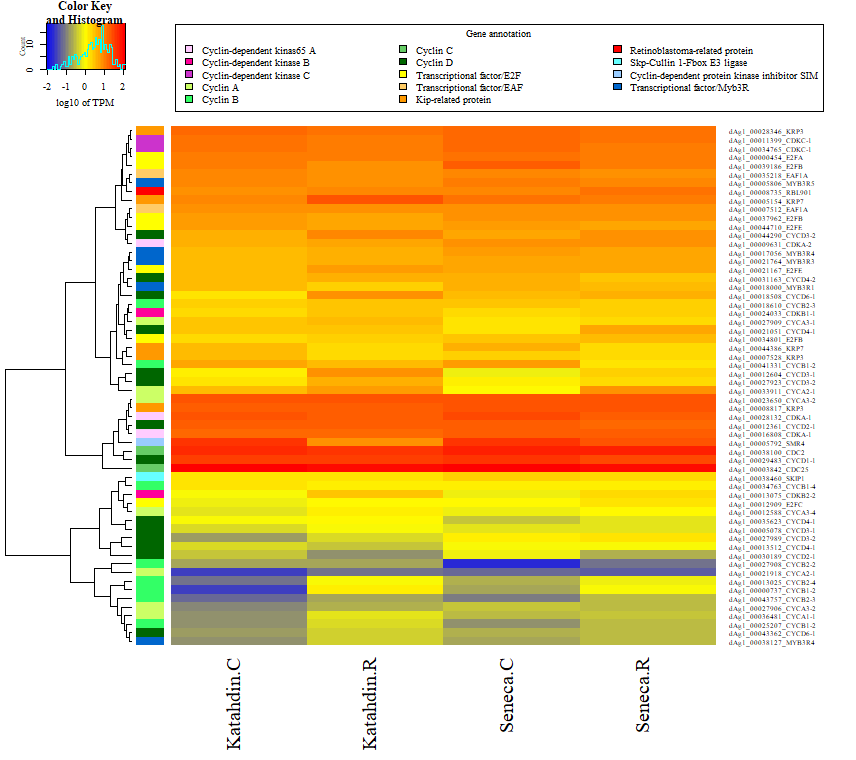


Figure S2. Heatmap of log10 of TPM values representing expression profiles of genes involved in the regulation of cell cycle progression in cultivars Katahdin and Seneca.
