# Supplementary material for: The Retinoblastoma-related gene RBL901 can trigger drought response actions in potato
Source: Plant Cell Rep. 2023 Aug 9;42(10):1701–4. doi: 10.1007/s00299-023-03055-0 (PMC10505103; doi:10.1007/s00299-023-03055-0)
Supplement: Supplementary file 3 — Supplementary file3 (DOCX 26 KB) [file 299_2023_3055_MOESM3_ESM.docx]

Supplementary Table S1. Analysis of variance in tuber yield parameters

| Factor | Df^1^ | MS^2^ | P-value |
| --- | --- | --- | --- |
| Tuber yield per plant (g/plant) | | | |
| (1) Cultivar | 5 | 529591 | 0,009 |
| (2) Treatment | 1 | 149511 | 0,301 |
| Interaction 1×2 | 5 | 91458 | 0,641 |
| Error | 24 | 134033 |  |
| Average tuber weight (g) | | | |
| (1) Cultivar | 5 | 2207,6 | 0,000 |
| (2) Treatment | 1 | 1010,8 | 0,005 |
| Interaction 1×2 | 5 | 506,1 | 0,003 |
| Error | 24 | 105,2 |  |
|  |  |  |  |

^1^ Degrees of Freedom (Df = n-1)

^2^ Mean square

n for “Cultivar” = 6

n for “Treatment” = 2 (control, recovery)

Table S2. Effect of drought on potato plants analyzed by JIP test parameters, calculated on the basis of fast fluorescence kinetics. The mean values of fluorescence parameters for cultivars under control and 3 days after drought release are presented; the p-value from two-way ANOVA was applied

| **Cultivar** | **Treatment** | **Fo** | **Fo/Fm** | **Fv/Fm** | **dVG/dto** | **dV/dto** | **ABS/RC** | **Dlo/RC** | **Tro/RC** | **ABS/Cso** | **Dlo/Cso** | **ABS/CSm** | **PI total** |
| --- | --- | --- | --- | --- | --- | --- | --- | --- | --- | --- | --- | --- | --- |
| Cayuga | Control | 415 | 0,1575 | 0,843 | 0,1743 | 0,3902 | 1,2211 | 0,193 | 0,917 | 415 | 65,431 | 2647 | 5,6409 |
|  | Recovery | 516 | 0,2041 | 0,796 | 0,3281 | 0,7165 | 1,5233 | 0,312 | 1,212 | 516 | 105,944 | 2540 | 1,4970 |
| Seneca | Control | 405 | 0,1534 | 0,847 | 0,1543 | 0,3549 | 1,1440 | 0,176 | 0,968 | 413 | 62,071 | 2640 | 3,7733 |
|  | Recovery | 511 | 0,2127 | 0,787 | 0,3387 | 0,7160 | 1,5605 | 0,339 | 1,222 | 511 | 109,459 | 2417 | 2,5456 |
| Kathadin | Control | 420 | 0,1523 | 0,848 | 0,1755 | 0,3999 | 1,2808 | 0,195 | 1,085 | 420 | 64,103 | 2763 | 3,9664 |
|  | Recovery | 465 | 0,1952 | 0,805 | 0,2612 | 0,5834 | 1,3312 | 0,261 | 1,070 | 419 | 185,238 | 2396 | 4,8374 |
| Dalila | Control | 404 | 0,1518 | 0,848 | 0,1611 | 0,3680 | 1,2353 | 0,188 | 1,048 | 404 | 61,378 | 2670 | 4,3357 |
|  | Recovery | 469 | 0,2020 | 0,798 | 0,2748 | 0,5962 | 1,4223 | 0,290 | 1,132 | 469 | 95,095 | 2333 | 1,7119 |
| Sebago | Control | 439 | 0,1616 | 0,838 | 0,1803 | 0,3981 | 1,2697 | 0,206 | 1,064 | 439 | 71,030 | 2726 | 2,9474 |
|  | Recovery | 515 | 0,2193 | 0,781 | 0,3547 | 0,7415 | 1,6330 | 0,366 | 1,270 | 515 | 114,158 | 2382 | 1,4738 |
| Pontiac | Control | 437 | 0,1725 | 0,827 | 0,2518 | 0,5761 | 1,4616 | 0,253 | 1,209 | 437 | 75,508 | 2547 | 2,1302 |
|  | Recovery | 618 | 0,2753 | 0,725 | 0,5774 | 1,1168 | 2,1722 | 0,577 | 1,540 | 618 | 178,412 | 2282 | 0,9942 |
|  |  |  |  |  |  |  |  |  |  |  |  |  |  |
| **Factor** | | **Statistics (p-value)** | | | | | | | | | | | |
| 1. Cultivar | | 0,000101 | 0,000003 | 0,000003 | 0,000000 | 0,000000 | 0,000000 | 0,000001 | 0,000002 | 0,000380 | 0,000004 | 0,000380 | 0,004020 |
| (2) Treatment | | 0,000000 | 0,000000 | 0,000000 | 0,000000 | 0,000000 | 0,000000 | 0,000000 | 0,000000 | 0,000000 | 0,000000 | 0,000000 | 0,000130 |
| (1x2) Cultivar x Treatment | | 0,012946 | 0,021498 | 0,022019 | 0,006411 | 0,032016 | 0,003984 | 0,003833 | 0,030801 | 0,010275 | 0,001840 | 0,010275 | 0,016293 |
